# A combination of low TMB and PD-L1 expression predict poor progression-free survival of metastatic melanoma patients treated with first-line ipilimumab plus nivolumab

**DOI:** 10.3389/fimmu.2026.1729883

**Published:** 2026-01-29

**Authors:** Baylor Akhavan, Wolfram Samlowski

**Affiliations:** 1 Kirk Kerkorian School of Medicine at UNLV, Las Vegas, NV, United States; 2 Nevada Oncology Specialists, Las Vegas, NV, United States; 3 University of Nevada School of Medicine, Reno, NV, United States

**Keywords:** BRAF mutation status, PD-L1 expression, progression-free survival, serum lactate dehydrogenase levels, tumor mutation burden

## Abstract

**Simple summary:**

Combinations of monoclonal antibodies that activate the immune system have been highly effective for treatment of melanoma that has spread (metastasized). Unfortunately, this type of immunotherapy treatment can trigger serious side effects. Thus, it would be valuable to predict patients who are unlikely to benefit from immunotherapy in advance. We evaluated four cancer-related markers to determine their usefulness. The level of two markers in cancer biopsies, PD-1 ligand (PD-L1) staining and tumor mutation burden (the number of mutations per megabase DNA), seemed to best predict treatment responses. In fact, patients who had low levels of both of these markers universally failed to respond to immunotherapy. Further work will be needed to develop computer tools to utilize these two markers to try to predict the potential usefulness of cancer immunotherapy in metastatic melanoma.

**Background:**

Combination checkpoint inhibitor therapy with ipilimumab plus nivolumab has significantly improved the treatment of patients with metastatic melanoma. This regimen has induced durable complete remissions and improved survival. However, these benefits are associated with a high risk of immune-related adverse events. We evaluated the usefulness of potential predictive biomarkers to determine which patients were unlikely to benefit from immunotherapy.

**Methods:**

A retrospective chart review was conducted of all metastatic melanoma patients treated by a single oncologist with ipilimumab plus nivolumab for advanced cutaneous or subungual melanoma. Baseline biomarkers including BRAF mutation status, serum lactate dehydrogenase levels, PD-L1 expression, and tumor mutation burden were correlated with progression-free survival (PFS).

**Results:**

Treatment outcomes were analyzed in 54 sequential patients. BRAF mutation status did not correlate with PFS. Only rare patients presented with an elevated lactate dehydrogenase (LDH); thus, this marker did not prove informative. There was a correlation of increased tumor mutation burden or PD-L1 expression with treatment response. Expression of either marker appeared to correlate with improved progression-free survival. An exploratory analysis suggested that the combination of low tumor cell PD-L1 expression and a low tumor mutation burden predicted an extremely poor immunotherapy response.

**Conclusions:**

Tumor mutation burden and PD-L1 represent potential predictive biomarkers for response to combination CKI therapy, while LDH and BRAF offer limited predictive value. Further work will be needed to develop a predictive nomogram to better aid in predicting potential immunotherapy benefit.

## Introduction

Melanoma is an aggressive form of skin cancer that has been steadily increasing in incidence in many parts of the world. From 2015 to 2019, the number of individuals who developed melanoma increased by 2-3% annually in the United States (US) (1). It has, therefore, been estimated that over 100,000 new cases of invasive melanoma will occur in the US in 2025, resulting in more than 8,000 deaths (1). Fortunately, there has been dramatic progress in the development of more effective melanoma treatments over the past 2 decades, including the discovery of targeted therapy as well immune checkpoint inhibitor (ICI)-based immunotherapy (2). These treatments have dramatically improved survival of patients with metastatic disease (3). The recent EA6134 DREAMseq trial firmly established combination immunotherapy as the preferred first line therapy for metastatic melanoma patients, in comparison to initial BRAF-mutation directed targeted therapy (4).

Combination immunotherapy utilizing antibodies directed against the inhibitory immune checkpoints PD-1 (nivolumab) and CTLA-4 (ipilimumab) has been shown to induce a high frequency of objective clinical responses. Dual checkpoint blockade further prolonged progression-free (PFS) and overall survival (OS), compared to single agent immunotherapy. With a median 10 years of follow-up, the combination regimen of nivolumab and ipilimumab achieved a remarkable median OS of 71.9 months (5). In contrast, the median survival was only 36.9 months following monotherapy with nivolumab and 19.9 months with ipilimumab alone. Overall, 31% of ipilimumab plus nivolumab treated patients achieved a durable complete remission and remained free of any recurrence by the end of the study (5). Notably, if patients were in remission at 3 years, the ten-year melanoma specific survival was 96% (5).

Even though ipilimumab plus nivolumab treatment improved outcomes for metastatic melanoma patients, this treatment resulted in substantial risks. Combination immunotherapy resulted in grade 3 or greater immune-related adverse events (irAEs) in at least 68.7% of patients (6). These side effects represent over-activation of the immune system and the development of autoimmunity. Commonly reported immunotherapy side effects include rash, colitis, hepatitis, and pneumonitis, but could possibly affect any other organ system (7). Most of these acute toxicities proved to be manageable with steroids and other immunosuppressive agents. Acute toxicities eventually improved following discontinuation of treatment (8–10). Unfortunately, there have been recent reports of long-term sequelae, such as endocrinopathy, arthropathy, and potentially accelerated atherosclerotic disease, that need to be considered when planning treatment for patients (11, 12).

Given the substantial risk of toxicity, it is important to define potentially responsive subsets of patients, who are the most likely to benefit from ICI therapy. In addition, it would also be helpful to identify poor risk patients, who are unlikely to benefit. A number of previously reported tumor-related biomarkers appeared to correlate with the outcome of melanoma treatment. These predictive biomarkers were generally identified from ICI monotherapy trials. Potential poor-risk markers included gene mutations at the BRAF V600 locus (13, 14), elevated serum lactate dehydrogenase (LDH) (15), a low percentage of tumor cells expressing programmed cell death protein 1 ligand (PD-L1) (16), as well as a low tumor mutation burden per megabase genomic DNA (TMB) (17). It is not clear how useful these predictive biomarkers are, given the expanded utilization of current combination ICI therapy with ipilimumab plus nivolumab.

The purpose of our study, therefore, was to evaluate the utility of these potential biomarkers as predictors of PFS in order to identify patients who were unlikely to benefit from ipilimumab plus nivolumab treatment.

## Materials and methods

### Study design

We performed a retrospective analysis of potential predictive biomarkers for combination immune checkpoint inhibition (ICI) therapy response. Potential subjects were identified by a computer search of a HIPAA compliant electronic patient care database (iKnowMed G2, McKesson, Houston, TX, US). The database was searched for all patients treated by a single physician (W.S.) with concurrent ipilimumab plus nivolumab between 2016 and 2024. Patients were eligible for analysis if they had unresectable, locally advanced or metastatic cutaneous melanoma (including acral or subungual disease). Patients were excluded if they had uveal, mucosal melanomas, or other cancers treated with combination ICI therapy. Patients who received only one dose of ICI therapy or were receiving 2^nd^ line or later therapy were also excluded. Patients who had received prior adjuvant single-agent ICI therapy for high-risk locoregional melanoma were also eligible for analysis.

### Data extraction

Each patient’s record was individually accessed. Relevant data were extracted and compiled in a password-protected Excel spreadsheet (Version 16.95 Microsoft Corporation, Redmond, WA, USA). A unique patient identification number was assigned to each patient. Age, primary diagnosis, site of metastases, and genetic mutations were recorded. The ICI treatment regimen, duration of therapy, patient outcome, adverse effects of immunotherapy, and current patient status were noted. Pre-treatment tumor cell PD-L1 expression was established based on immunostains of core needle biopsies of metastatic lesions using 22C3 monoclonal antibody. Tumor mutation burden (TMB, expressed as mutations/Mb) was calculated by sequencing formalin-fixed, paraffin embedded tumor tissues obtained by biopsy of metastatic lesions via commercial Next Gen sequencing (Tempus, Chicago, IL, USA or Foundation Medicine, Cambridge<MA, USA). Pretreatment serum LDH (obtained within 2 weeks prior to starting therapy, units/L) was also recorded. Following data extraction, patient identifiers were removed. This study design was reviewed by the Western IRB chair and deemed exempt from full board review.

### Treatment regimens

Patients were treated with 4 doses of either the standard induction regimen (ipilimumab 3 mg/kg plus nivolumab 1 mg/kg every 3 weeks I.V.) (6), or the alternate regimen (“flipped dose” regimen; ipilimumab 1 mg/kg plus nivolumab 3 mg/kg every 3 weeks I.V.) (18). After completion of induction therapy, maintenance treatment with a fixed dose of 480 mg nivolumab I.V. monthly was continued in responding patients. Patients who achieved a confirmed complete remission were considered for elective treatment discontinuation following an institutional protocol (19).

### Evaluation of outcomes

The best objective response (BOR) to therapy at 12 months was utilized to determine individual patient response to treatment based on RECIST 1.1 criteria (20). A patient achieved a complete response (CR) if they had resolution of all known sites of disease. A partial response (PR) was characterized by an over 30% decrease in the sum of bidimensional measurements of index lesions. Progressive disease (PD) resulted from a greater than 20% increase in the sum of bidimensional tumor dimensions. Patients who did not achieve CR, PR or PD were characterized as having stable disease (SD).

### Statistical analyses

Descriptive statistics, including range, median, standard deviation, were calculated with the use of the Excel spreadsheet. The data of last clinic follow-up, progression or death was used to calculate PFS and OS. Responding patients were censored at the date of last follow-up. PFS and OS were calculated from the start of immunotherapy via Kaplan Meier analysis, performed via GraphPad Prism software (v10.6.1) (21). A log-rank test was used to compare paired progression-free survival curves (22). The Kruskal Wallis test was used to compare multiple survival curves (23). The date of the final analysis was November 25th, 2024.

## Results

### Demographics

A total of 54 patients diagnosed with advanced or metastatic cutaneous melanoma underwent first line therapy ICI therapy with ipilimumab plus nivolumab. There were 51 patients (94.4%) with cutaneous melanoma and 3 patients (5.6%) with acral or subungual melanoma. A total of 34 patients were men (63.0%) and 20 were women (37.0%). The median age at diagnosis was 63 ± 15 years (± standard deviation), with a range from 20 to 92 years. Forty-nine patients were Caucasian (90.7%). There were four Hispanic individuals (7.4%), and one patient of Asian descent (1.9%). Individual descriptions of patient demographics are provided (**Supplementary Table S1**).

### Characterization of potential “driver” mutation profile

Tumor cell expression of non-overlapping “driver” mutations was evaluated by Next Gen sequencing. This testing revealed that 20 patients had a BRAF V600 mutation (37.0%), 14 with a BRAF V600E mutation and 6 with a BRAF V600K mutation. An additional 19 patients had RAS mutations (35.2%), and 5 patients had a NF1 mutation (9.3%). Two patients had a C-KIT mutation (3.7%), and eight patients were “quadruple negative” (no BRAF, RAS, NF-1 or C-KIT mutations) (14.8%).

### Treatment outcome

Of our 54 patients, 18 (33.3%) received the standard treatment regimen of 3 mg/kg ipilimumab and 1 mg/kg nivolumab, while the remaining 36 patients (66.7%) received the alternate (or “flipped”) regimen of ipilimumab plus nivolumab (18). The PFS of the standard and alternate treatment regimens was not statistically different, so results were pooled for analysis (data not shown). The median potential follow-up of our patients was 48.5 ± 22.6 months. For the entire cohort of patients, median PFS was indeterminate and appeared to plateau after 2 years. At 4 years of follow-up, 51.8% of patients in the entire cohort remained progression-free (**Supplementary Figure S1A**). The median OS also remained undefined (**Supplementary Figure S1B**), with 59.8% of patients alive at 4 years. Individual descriptions of patient outcome are provided (**Supplementary Table S2**).

### Analysis of potential predictive markers for ipilimumab/nivolumab response

We evaluated whether commonly utilized risk factors, such as BRAF V600 mutations, serum LDH, PD-L1 expression, and TMB, predicted response to subsequent combination ICI treatment in our patient population.

**Figure 1:**
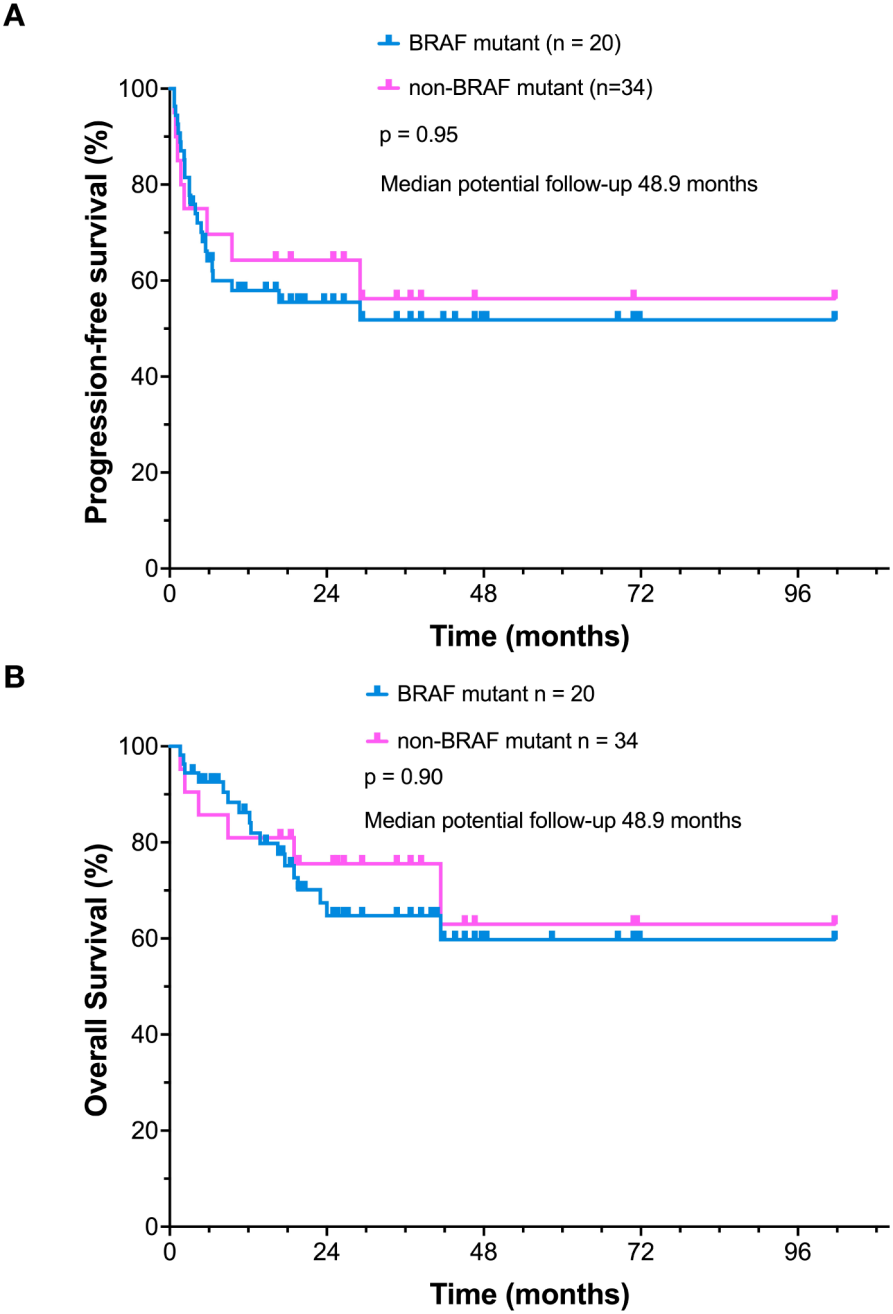
Kaplan–Meier survival analyses. **(A)** Comparison of PFS between patients with BRAF-mutated and tumors without a BRAF V600 mutation. **(B)** Comparison of OS between patients with BRAF-mutated and tumors without a BRAF V600 mutation.

BRAF mutations are thought to associate with more aggressive clinical behavior of melanoma (14). We evaluated whether patients with BRAF mutations had adverse clinical outcome following combined ipilimumab plus nivolumab therapy. Subgroup analysis based on BRAF mutation status demonstrated that PFS of patients with BRAF mutations versus those with other “driver” mutations (NRAS, NF-1, C-KIT or quadruple negative) was similar following ipilimumab plus nivolumab treatment (**Figure 1A**) (p=0.95). There were no detectable differences in OS between groups of patients with and without BRAF mutations (**Figure 1B**) (p=0.90). In subsequent biomarker analyses, we therefore focused on PFS as a surrogate for long-term outcome. Mutation-specific PFS and OS for each additional mutation is also provided (**Supplementary Figures S2A, B**).

Elevated pre-treatment serum LDH levels have been proposed to identify patients who are less likely to benefit from ICI therapy (13, 15, 24). Pretreatment serum LDH was measured at baseline, prior to initiation of combination immune checkpoint inhibitor therapy, This data was available for all 54 patients. The median pre-treatment LDH level was 187 ± 87 U/L (normal 120–250 U/L). In our patient population, patients presenting with an elevated LDH were infrequent. Only 8 patients (18.5%) exhibited elevated LDH levels (≥ 250 U/L).

**Figure 2:**
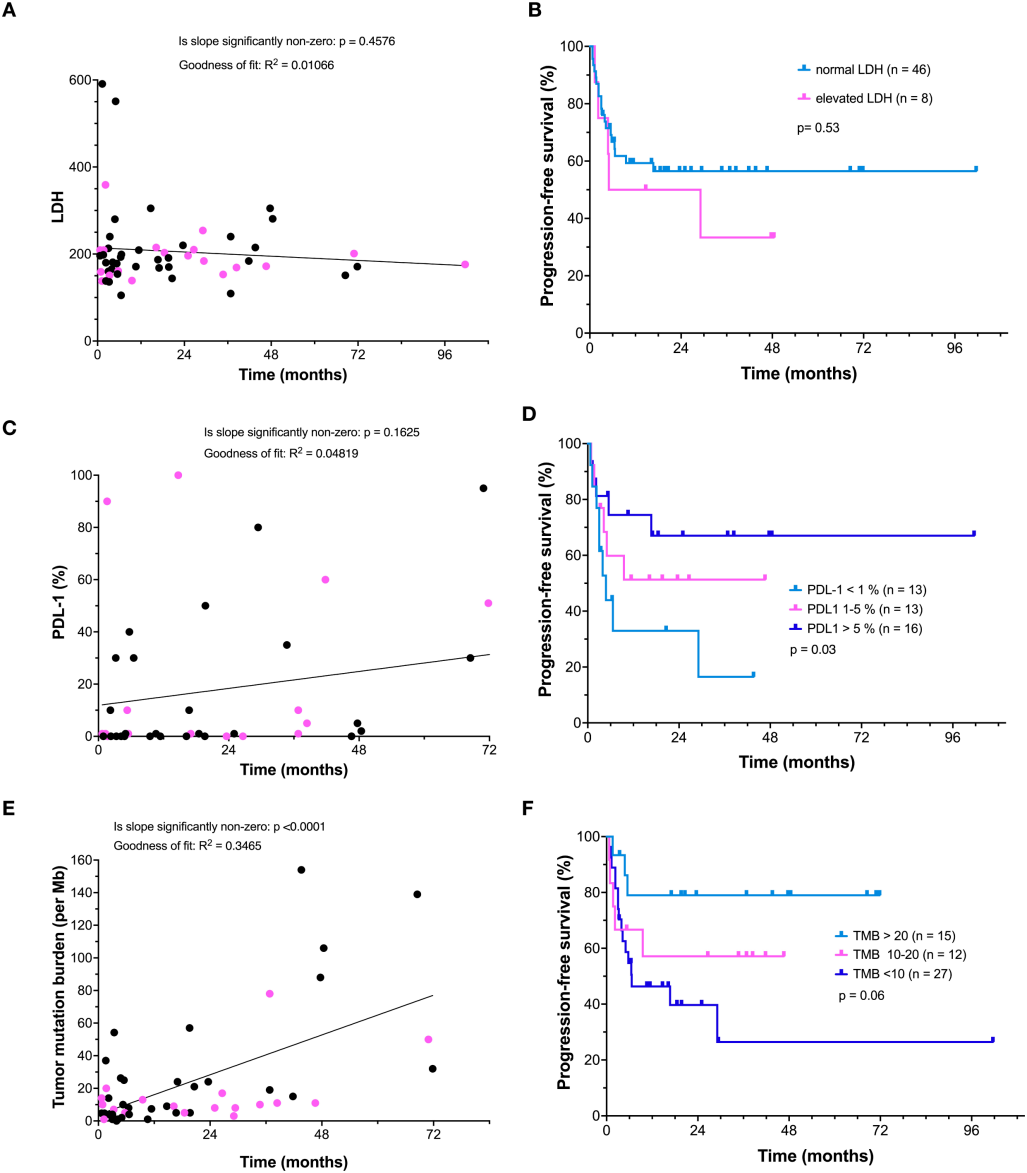
Associations between biomarkers and progression-free survival (PFS). **(A)** Correlation between pre-treatment lactate dehydrogenase (LDH) levels (IU/ml) and PFS duration. Tumors with a BRAF mutation are indicated (pink) **(B)** Kaplan–Meier analysis comparing PFS in patients with elevated versus normal LDH. **(C)** Correlation between PD-L1 expression and PFS is shown, BRAF-V600 mutated (pink) tumors and tumors without a BRAF V600 mutation (black) are indicated. **(D)** Kaplan–Meier analysis of PFS stratified by indicated PD-L1 expression levels. **(E)** Correlation between tumor mutational burden (TMB) and PFS. BRAF-V600 mutated (pink) tumors and tumors without a BRAF V600 mutation (black) are indicated. **(F)** Kaplan–Meier analysis of PFS stratified by indicated TMB levels.

We evaluated whether pre-treatment LDH levels correlated with outcome. A scatter plot directly comparing pre-treatment LDH with the duration of PFS following treatment is shown (**Figure 2A**). There was minimal correlation of numerical LDH value and PFS. Only 2 of the 20 patients with a BRAF mutation had an elevated LDH.

Progression-free survival in patients with a normal (n=44) versus an elevated LDH (n=8) was also evaluated by Kaplan-Meier analysis (**Figure 2B**). Although there was a trend toward inferior outcome in patient with elevated LDH, this did not reach statistical significance (p=0.53), perhaps due to the limited number of patients who presented with an elevated LDH in our clinical practice. In addition, the limited number of patients with elevated LDH did not warrant further exploration with correlation to BRAF mutants and treatment outcomes for a meaningful comparison.

PD-L1 expression on tumor cells has been correlated with immunotherapy response in numerous tumor types, including melanoma (16, 25). PD-L1 testing was not considered standard-of-care over the interval of this study. Thus, PD-L1 tumor proportion scores (TPS) were available for only 42 of our patients (77.8%). Twenty-nine of 42 patients (69.1%) had increased PD-L1 expression (TPS ≥ 1%), 19 had over 5% PS-L1 expression. Thirteen patients (30.9%) had minimal PD-L1 expression (TPS < 1%).

A plot of the percentage of tumor cells expressing PD-L1 showed a modest linear correlation with PFS (**Figure 2C**). While BRAF mutant patients tended to have lower PD-1 ligand scores, this was not associated with a worse outcome.

PFS was also stratified by PD-L1 expression using empirically selected cutoff values to ensure adequate group sizes for comparison (**Figure 2D**). Generally, PD-L1 expression over 1% is accepted as clinically significant (16). The poorest outcomes were observed in patients with PD-L1 <1%, where only 16.5% remained progression-free at 36 months (p=0.03 by log-rank test). However, in our patient series, tumors with PD-L1 expression >5% experienced the most favorable outcomes, with 67.0% remaining progression-free at 36 months. Those with PD-L1 scores between 1–5% showed moderately improved outcomes, with 51.3% progression-free at 36 months.

Tumor mutation burden is also thought to correlate with ICI treatment outcome in many cancers, including melanoma (17, 25). A TMB of 10 mutations/Mb has been associated with an increased likelihood of PD-1 monotherapy response (17). TMB data was available for 52 of our patients (96.3%). TMB assessment was indeterminant in the other two individuals. Thirty-seven of 52 patients (71.2%) had a TMB score ≥ 10 mutations/Mb, and 15 patients (28.8%) had a TMB score < 10 mutations/Mb.

We evaluated the effect of tumor mutation burden on PFS in our patients treated with combination therapy. TMB was plotted versus PFS. This analysis revealed a significant linear correlation between an elevated TMB and prolonged PFS (**Figure 2E**). While patients with BRAF mutations tended to have a lower tumor mutation burden, a relationship to worsened PFS was not apparent.

PFS was also plotted based on empirically selected TMB cutoff values intended to ensure sufficient sample sizes for meaningful comparison (**Figure 2F**). Patients with a low TMB (<10 mutations/Mb) had a relatively poor outcome with only 26.5% of these patients remaining progression-free at 36 months. In patients with an intermediate TMB between 10–20 mutations/Mb, 57.1% were progression-free at the same time point (p=0.06). In contrast, patients with TMB >20 mutations/Mb demonstrated the most favorable outcomes, with 79.0% remaining progression-free at 36 months.

**Figure 3:**
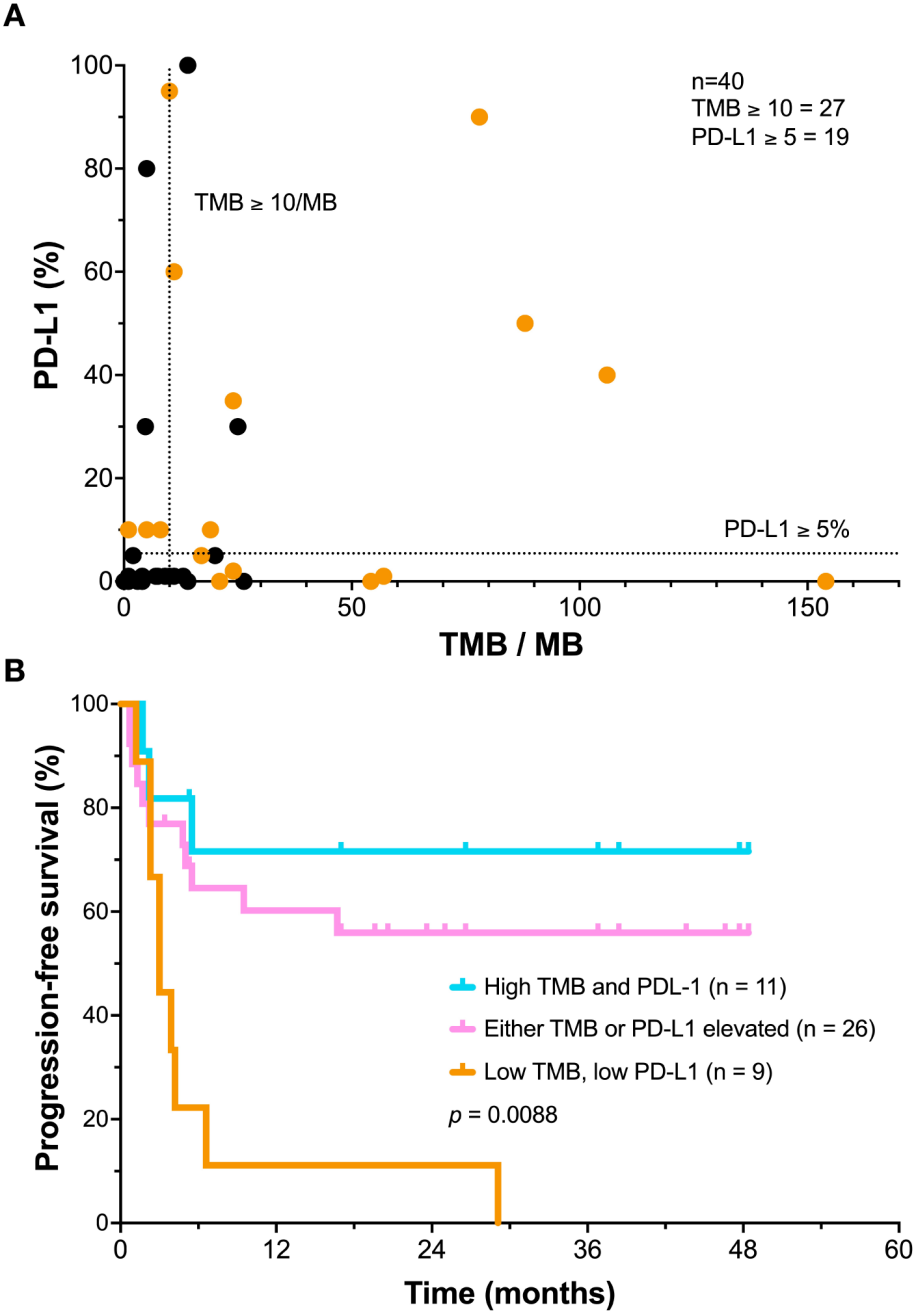
Exploratory analysis of the relationship between TMB and PD-1 ligand expression and PFS after combination immunotherapy. **(A)** Each patient’s TMB was plotted versus PD-1L score. Patients with durable progression-free survival are indicated in orange. The TMB score cutoff (10/MB) and PD-L1 score (5%) were indicated by dashed lines. **(B)** Kaplan–Meier analysis of progression-free survival (PFS) in the exploratory subgroup of patients stratified by both low tumor mutational burden (TMB <10/MB) and low PD-L1 expression (<1%), compared to patients with either TMB ≥ 10 or PD-L1 ≥ 5, or with increased levels of both markers. Comparison of the outcomes by log rank test demonstrated a *p* value of p=0.0088.

A further exploratory analysis of the distribution of TMB versus PD-L1 expression was performed in patients where both biomarkers were available. Patients with durable progression-free survival are indicated (**Figure 3A**). This analysis showed that patients with either increased TMB or PD-L1 expression appeared to benefit from combination immunotherapy (n=28). The key highlight of this graph demonstrates that patients who expressed low level of both markers (n=10) all failed to achieve durable responses following combination immunotherapy (**Figure 3A**).

A Kaplan Meier analysis of progression-free survival based on this dataset was performed. Patients with a PD-L1<5% and TMB < 10/MB (n=9) had a brief median PFS following ipilimumab plus nivolumab therapy of only 3.5 ± 8.2 months (**Figure 3B**). All patients with both low PD-L1 and TMB rapidly died due to metastatic disease. In contrast, patients who had increased levels of either TMB or PD-L1 had a median PFS of 16.8 ± 16.4 months. Most of these responses proved durable. Patients who exhibited increased expression of both markers (n=11) appeared to have a further improvement in PFS (26.6 ± 18.5 months), These differences appeared significant (*p* = 0.0088) by Kruskal-Wallis test.

## Discussion

Following the advent of immune checkpoint inhibitor therapy, the prognosis of patients with metastatic melanoma has improved significantly. A number of potential tumor-specific predictive markers for melanoma have previously been identified for patients with metastatic melanoma, including BRAF V600E mutation status, LDH, PD-L1, and TMB. However, the usefulness of these factors in predicting the outcome of ipilimumab and nivolumab combination therapy administered in a community setting remains to be established. We therefore performed a retrospective review of treatment data from 54 sequentially treated patients who received ipilimumab plus nivolumab as their initial therapy for metastatic melanoma. It should be noted that additional host-specific prognostic biomarkers have been proposed, including T-cell infiltration into tumors (26, 27), as well as tumor immunoscore (28, 29). Since these biomarkers were not routinely quantified in our patients, these markers were not evaluated in our current study.

The goal of the study was to evaluate whether potential biomarkers, evaluated at the time of diagnosis, had predictive significance. Progression-free survival was utilized as the primary endpoint, as patients currently can have significant clinical benefit from 2^nd^ or 3^rd^ line treatment, which is likely to confound assessment of overall survival.

BRAF V600 mutation status has historically been associated with decreased recurrence-free survival (RFS) and disease-specific survival (DSS) (30). Patients with resected stage IIIB and IIIC melanoma with a BRAF V600 mutation had an increased risk of recurrence and metastases (14). In the Checkmate-067 trial, patients with BRAF mutations had an inferior outcome following checkpoint inhibitor monotherapy, compared to dual ICI treatment (13). Our current patient series demonstrated that the presence or absence of BRAF V600 mutations had minimal effect on progression-free and overall survival in patients receiving combination ipilimumab plus nivolumab therapy.

In addition to BRAF mutations, serum lactate dehydrogenase (LDH) has long been recognized as an adverse predictive biomarker in metastatic melanoma (31). Elevated LDH levels are typically correlated with more aggressive disease kinetics. Yet, it is currently rare in our clinic for patients with newly diagnosed melanoma to present with an elevated LDH at the start of therapy. We could only identify 8 patients in our entire patient series who had an elevated LDH at baseline. The low frequency of LDH elevations seen in our study is consistent with similar trends reported by Gershenwald et al. (32). Long et al. found that the effectiveness of ipilimumab and nivolumab immunotherapy was markedly reduced in patients with an elevated LDH (13). However, in our patient series, the low prevalence of LDH elevation limited its utility as a biomarker for dual immunotherapy response. There was, at best, a weak association with PFS, that did not reach statistical significance. Thus, serum LDH was not a practical marker for predicting progression-free survival in our patients.

We believe that the decreasing frequency of patients with elevated LDH may be the result of more diligent follow-up of high-risk patients. Ultimately, there appears to be a trend toward earlier detection and treatment of metastatic disease. Currently, only rare patients present with a massive disease burden and high LDH. This finding may also reflect changes in referral patterns, sites of metastatic disease, as well as the effects of previous treatment (e.g., adjuvant immunotherapy).

Other tumor biomarkers, such as TMB and PD-L1, both had a strong correlation with objective responses following PD-1/PD-L1 monotherapy across multiple tumor types (25, 33). PD-L1 expression and TMB are independent predictive factors in most cancers evaluated to date (25). The impact of TMB and PD-L1 scores on the potential outcome of combination immunotherapy in metastatic melanoma is not yet fully understood.

Patients with elevated PD-L1 expression on tumor cells have an increased responsiveness to ICI therapy across a broad variety of cancers (25, 33). It should be noted that PD-L1 expression on melanoma tumor cells is generally lower than is observed in other cancers, such as NSCLC or renal cell carcinoma (34). As a result, there is some disagreement concerning the degree of correlation of PD-L1 expression on tumor cells with clinical responses (35, 36).

Our analysis demonstrated a significant relationship of increasing levels of PD-L1 expression and progression-free survival following first-line therapy. Patients with PD-L1 <1% on tumor cells had an estimated 4-year progression-free survival of 16%. PFS was 51% in patients with 1-5% PD-L1 expression and 67% in patients with PD-L1 >5%. In our patient series, patients with BRAF mutations trended to be associated with lower tumor cell PD-L1 expression, however this did not appear to adversely affect outcomes. It should also be noted that low PD-L1 levels (<1%) did not preclude durable responses to dual checkpoint inhibition.

In a separate retrospective analysis, increased TMB (>10 mutations/MB) also identified a subgroup of patients across multiple tumor types who generated a robust tumor response following pembrolizumab monotherapy (17). This observation was subsequently confirmed in 8000 patients with multiple cancer types treated with PD-1/PD-L1 antibodies. Based on this data, Gandara et al., proposed a TMB cutoff >10 mutations per megabase, as this correlated significantly with OS (37). This cut-off has subsequently been widely employed in clinical trials.

Generally, in metastatic melanoma patients, an increased TMB was found to be associated with improved survival, if a BRAF mutation was not present (38). Andrews et al. subsequently evaluated a cohort of deidentified melanoma patients from a large clinico-genomic database (39). These patients all had comprehensive genomic profiling and TMB scoring. Patients were treated with either PD-1 monotherapy or combination immunotherapy. TMB-high status (≥10 mutations per megabase) was independently predictive of superior progression-free survival and overall survival in both mono and combination ICI therapy patients. In another melanoma-specific study, elevated TMB was associated with significantly longer progression-free survival following dual-agent ICI therapy (HR 0.26, 95% CI 0.07-0.90, p = 0.033, log-rank test) (40, 41). In contrast, a meta-analysis by Ning et. al., found that patients with high TMB showed significantly improved OS and PFS following PD-1 monotherapy (42). However, this increased benefit was not observed in patients receiving combination immunotherapy in the same report (42). Thus, measurement of PD-L1 and TMB as predictive markers in dual checkpoint inhibitor therapy for metastatic melanoma is not a current standard-of-care.

Within our cohort of metastatic melanoma patients, we also identified a strong correlation of TMB with long-term PFS. In patients with TMB <10/MB, 48 month estimated progression-free survival was 26%, which increased to 57% in patients with TMB 10-20/MB and 79% if TMB was >20/MB. It should be noted that durable responses were seen at all levels of TMB expression. Thus, TMB alone was not sufficient to identify a group of patients who would fail to respond to combination ICI therapy. It should also be noted that the optimal cut-points for either PD-L1 or TMB as a predictive markers have not been established to date (43). Optimal cut-off values are likely to differ in various tumor types (43).

Considering the apparent usefulness of PD-1 ligand and TMB scores in our patient cohort, we performed an exploratory analysis of the uncommon patients that had both PD-L1 <5% and TMB <10/Mb compared to the rest of the cohort. Despite a small sample size (9 patients), we found that these patients had an extremely poor outcome and were unlikely to respond to combination checkpoint inhibitor therapy. We believe that it is important to identify these high-risk patients as candidates for novel treatment options and further research.

Potential limitations of our study include a small sample size and uncertainty surrounding the optimal cutoff values for PD-L1 and TMB. Due to the limited cohort size, threshold values for these biomarkers were selected to preserve statistical power and allow for meaningful subgroup comparisons. Additionally, it should be noted that PD-L1 testing was not routinely performed as part of standard clinical care. These factors restricted the number of evaluable patients, limiting our ability to fully evaluate the predictive impact of this test. Thus, our data should be considered exploratory and in need of confirmation in a larger series of patients. Furthermore, while our analysis focused on key biomarkers, this approach excluded a more comprehensive evaluation of other potentially relevant biomarkers. Finally, the number of patients with both a low TMB and PD-L1 score were quite small. The markedly adverse impact of this combination of biomarkers will also require further evaluation in a larger patient series.

## Conclusions

While BRAF mutation status and serum LDH have shown potential predictive significance in previous trials, these markers were not informative in our patient population. Our findings suggest that increasing PD-L1 expression and tumor mutational burden (TMB) were individually associated with improved outcomes in patients receiving dual immune checkpoint inhibitor therapy for advanced melanoma. The combined testing of PD-L1 and TMB may offer a more nuanced approach to patient stratification, particularly in identifying individuals who may derive limited benefit from current therapies or those that could be prioritized for novel treatment strategies or clinical trials. Patients with both low TMB and PD-L1 expression in tumors do not appear to benefit from combination ipilimumab plus nivolumab therapy. Thus, these patients should be considered for clinical trials of novel agents. Future prospective studies with larger, more diverse populations are needed to validate optimal biomarker thresholds and to generate a predictive nomogram that can more accurately guide discussions of potential clinical benefit versus toxicity risks in metastatic melanoma patients.

## Data Availability

The original contributions presented in the study are included in the article/**Supplementary Material**. Further inquiries can be directed to the corresponding author.

## Supplementary material

Supplementary Figure 1 **(A)** Kaplan Meier analysis of progression-free survival (PFS) in the overall cohort. **(B)** Overall survival (OS) in the overall cohort.

Supplementary Figure 2 **(A)** Kaplan Meier analysis of progression-free survival (PFS) based on genotype (including non-BRAF mutations). **(B)** Overall survival (OS) based on genotype (including non-BRAF mutations).
