# Supplementary figures and images for: A combination of low TMB and PD-L1 expression predict poor progression-free survival of metastatic melanoma patients treated with first-line ipilimumab plus nivolumab
Source: Front Immunol. 2026 Jan 29;17:1729883. doi: 10.3389/fimmu.2026.1729883 (PMC12894002; doi:10.3389/fimmu.2026.1729883)

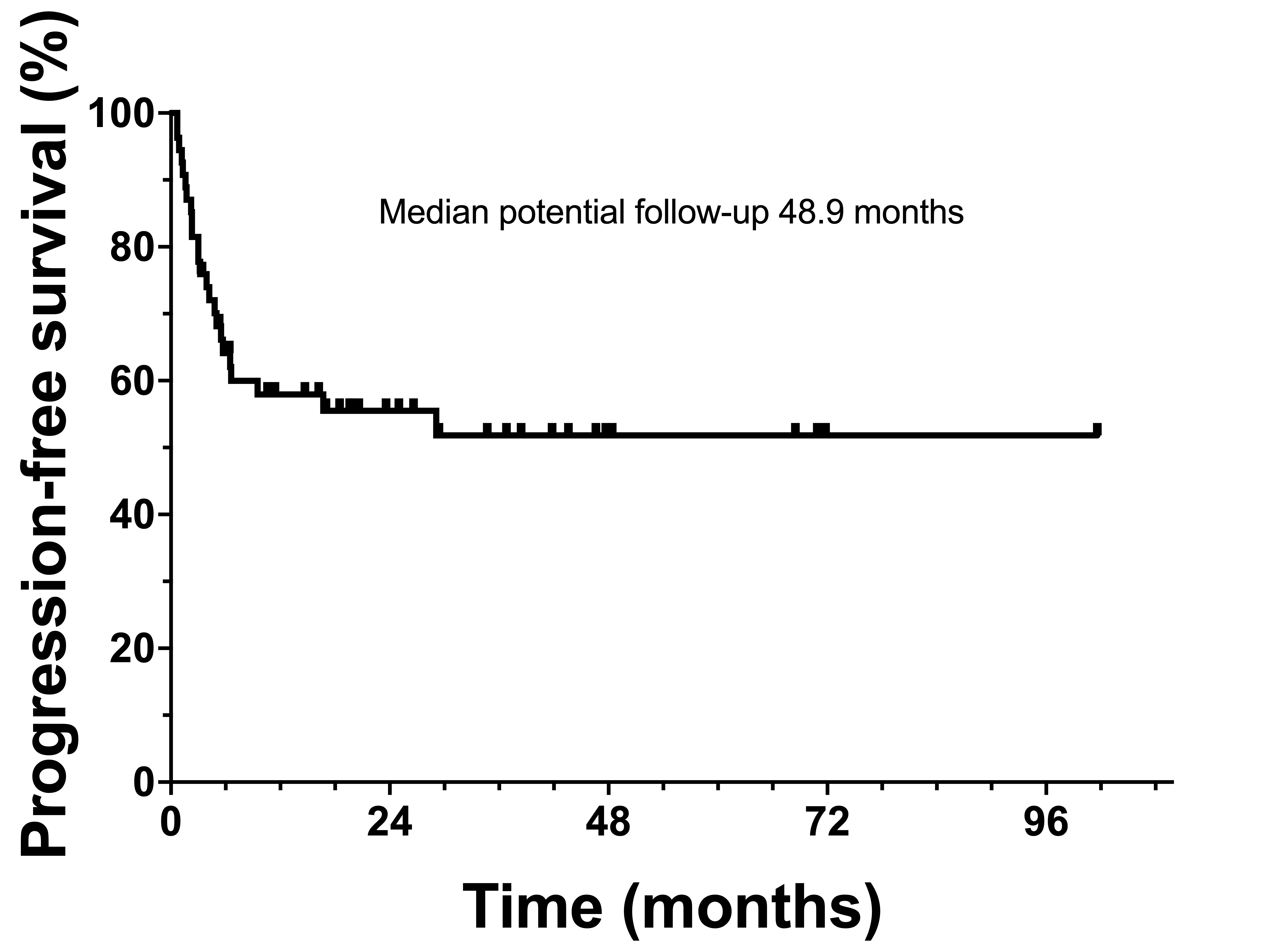

Supplement: Supplementary Figure 1 — (A) Kaplan Meier analysis of progression-free survival (PFS) in the overall cohort. (B) Overall survival (OS) in the overall cohort. [file Image1.jpeg]

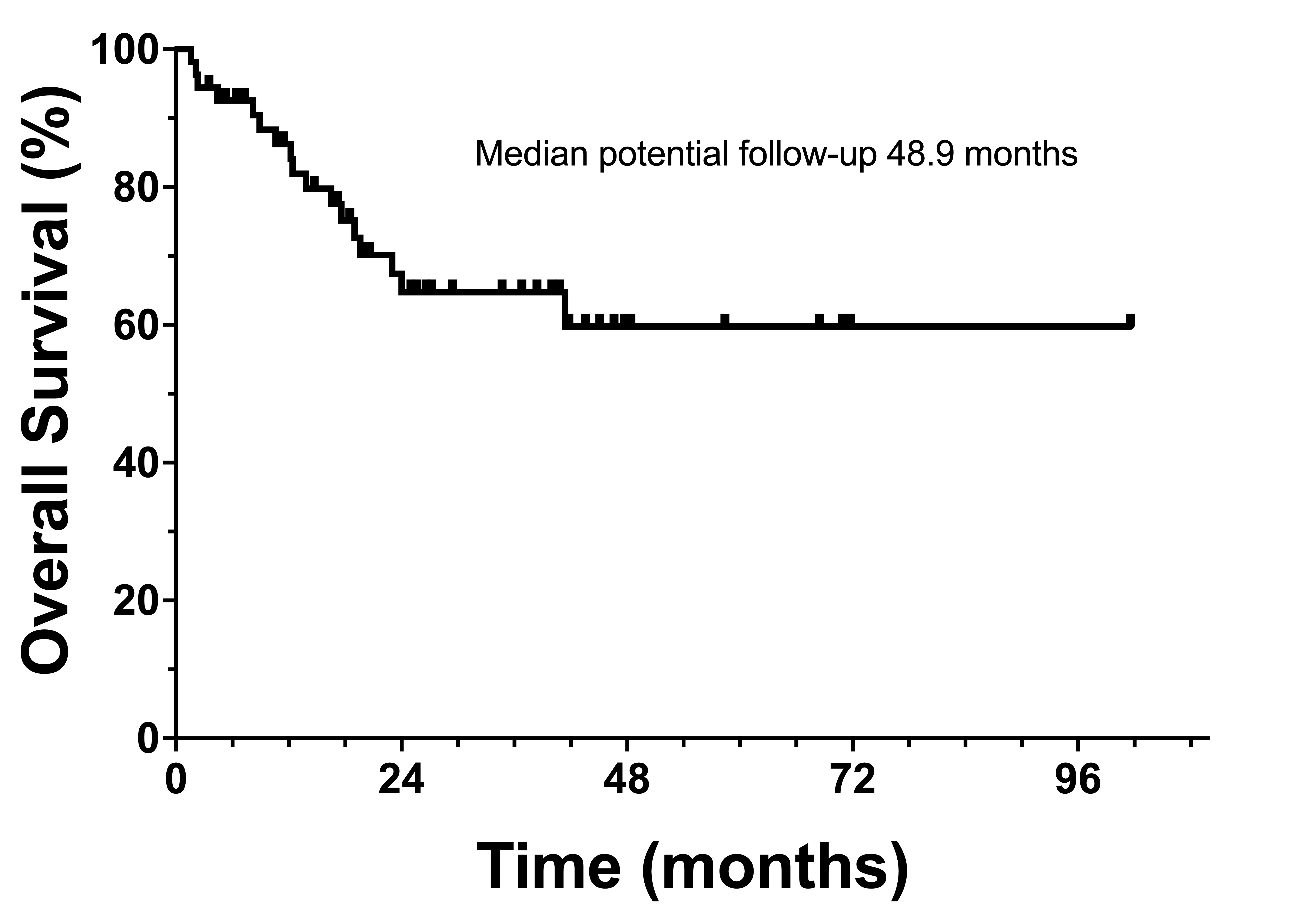

Supplement: Supplementary Figure 2 — (A) Kaplan Meier analysis of progression-free survival (PFS) based on genotype (including non-BRAF mutations). (B) Overall survival (OS) based on genotype (including non-BRAF mutations). [file Image2.jpeg]

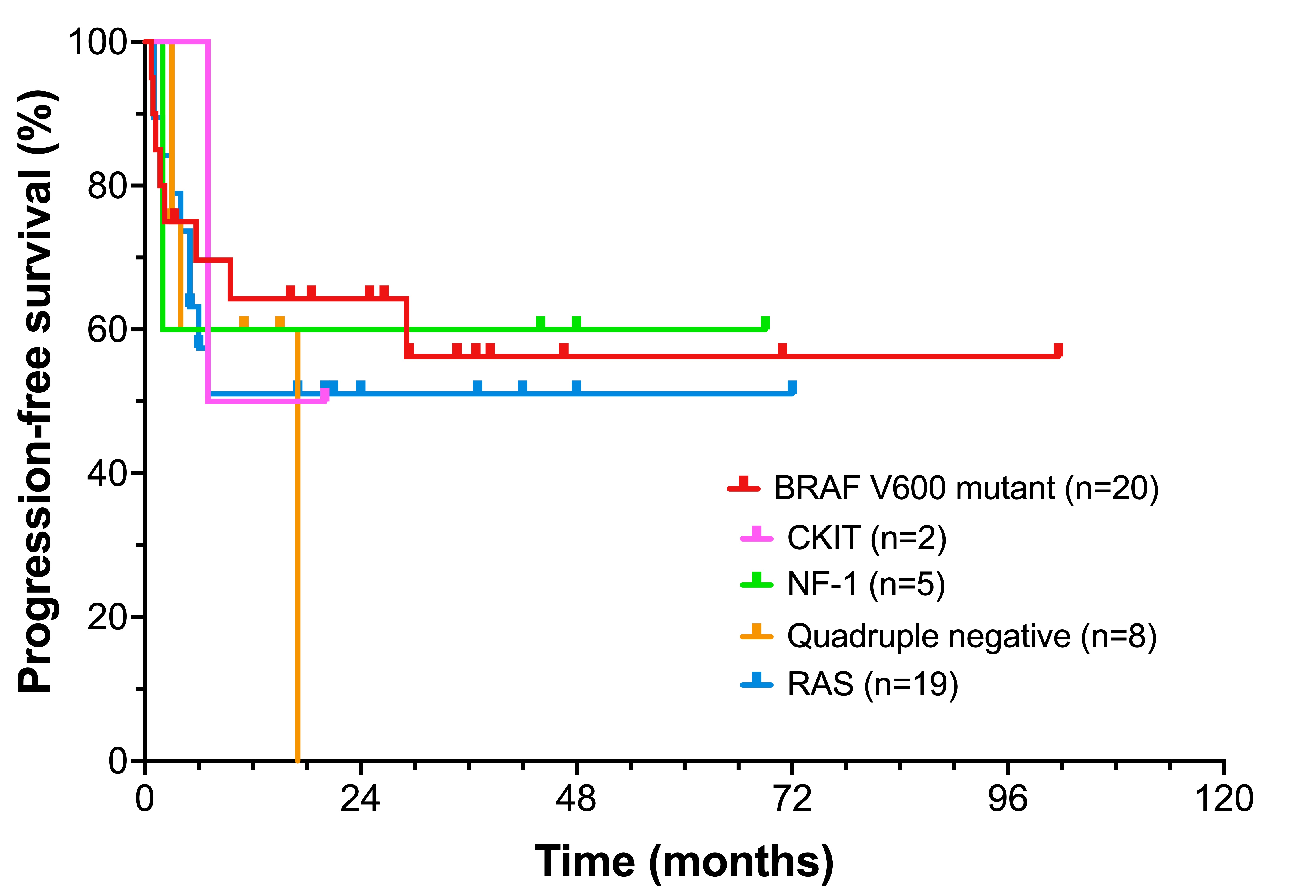

Supplement: Supplementary file 3 [file Image3.jpeg]

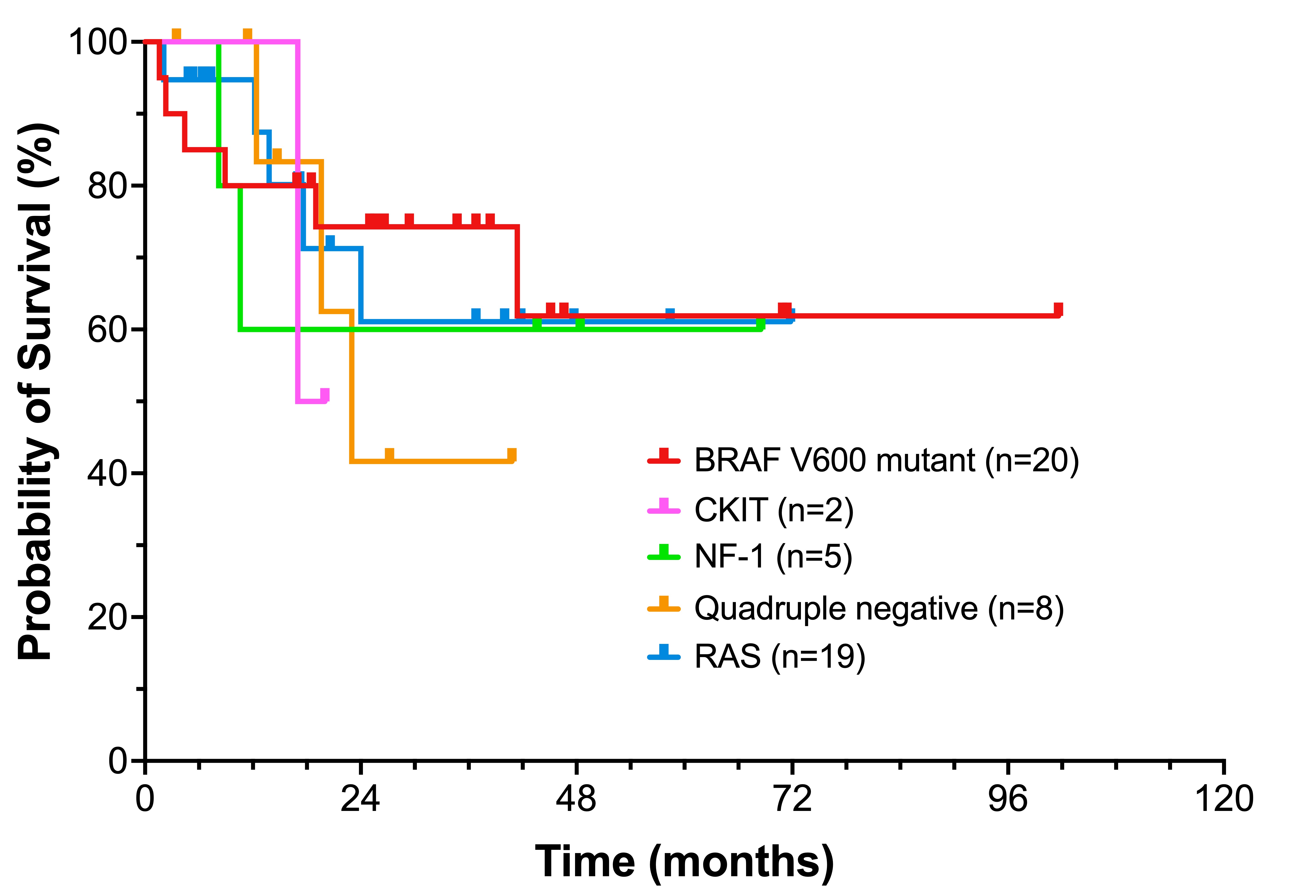

Supplement: Supplementary file 4 [file Image4.jpeg]
