# Supplementary material for: A combination of low TMB and PD-L1 expression predict poor progression-free survival of metastatic melanoma patients treated with first-line ipilimumab plus nivolumab
Source: Front Immunol. 2026 Jan 29;17:1729883. doi: 10.3389/fimmu.2026.1729883 (PMC12894002; doi:10.3389/fimmu.2026.1729883)
Supplement: Supplementary file 5 [file Table1.docx]

**Table 1: Patient demographics**

| **Pt** | **Mutation** | **Age** | **Sex** | **Race** | **Primary site** | **Site of metastases** | **Initial LDH** | **TMB (/Mb)** | **PDL1**  **(%)** | **Comorbidities** |
| --- | --- | --- | --- | --- | --- | --- | --- | --- | --- | --- |
| 1 | QN | 64 | F | C | Upper Limb | Left axilla | 209 | 7 | 1 | hypothyroidism, Anxiety |
| 2 | NRAS Q61K | 69 | M | C | Lower Limb | LNs (ABD, groin) | 180 | 4 | 1 | HTN, CAD, TIIDM, |
| 3 | NRAS Q61L | 56 | M | C | Axilla | LNs (retroperitoneal) | 171 | 32 | N/A | ulcerative colitis, TIIDM, peripheral neuropathy, hypothyroidism, HTN |
| 4 | QN | 68 | F | C | Head | LNs (ABD), bone (left sacral) | 187 | 5 | 80 | N/A |
| 5 | KRAS G12D, NF1 R440*, NF1 splice site (5609+1G>A) | 44 | M | C | Trunk | LNs (left axilla) | 178 | 10 | 95 | ulcerative enterocolitis |
| 6 | BRAF V600E | 41 | F | C | Face | LNs (cervical) | 151 | 7 | 1 | hypothyroidism |
| 7 | BRAF V600E | 58 | M | C | Trunk | Bilateral lung, left rib | 254 | 3 | 0 | ulcerative colitis, anxiety, hypogonadism, arthritis, HTN, hypogonadism, gynecomastia, hypercholesterolemia |
| 8 | BRAF V600E | 75 | F | C | Upper Limb | Lung, LNs (right axilla) | 209 | 14 | 0 | RA |
| 9 | BRAF V600K, NRAS G60R | 68 | M | C | Trunk | Right lung, left paraspinal muscle, right breast | 201 | 50 | N/A | AF, HTN, Prostate Cancer, Actinic keratosis |
| 10 | NRAS Q61L | 65 | M | C | Trunk | Lung, LNs (left axilla) | 154 | 25 | 30 | Ulcerative colitis, pulmonary embolism, panhypopituitarism, DVT, hypothyroidism, sleep apnea |
| 11 | HRAS G13R | 72 | F | C | Lower Limb | SQ (breast) | 220 | 24 | 2 | Psoriasis vulgaris, pneumonitis, pulmonary fibrosis, HTN |
| 12 | BRAF V600E | 55 | M | C | Trunk | SQ, peripancreatic, bone | 215 | 9 | 1 | CAD, hypercholesterolemia, TIIDM, chronic inflammatory demyelinating polyradiculoneuropathy (CIDP), MI, HTN |
| 13 | NF1 R440*, NF1 Q1174* | 79 | M | C | Head | LNs (cervical) | 215 | 154 | 0 | Aortic aneurysm, hyperlipidemia, arthritis, psoriasis, HTN, appendectomy |
| 14 | QN | 73 | M | C | Lower Limb | N/A | 240 | 54 | 0 | HTN, DVT, Crohn’s Disease, Plaque psoriasis, Hyperlipidemia, TIIDM, neuropathy, anemia |
| 15 | Nonfunctional BRAF, NF1 splice site (c.2990+2T>A), R1276* | 73 | F | H | Upper Limb | Lung | 198 | 37 | N/A | Anemia, non-Hodgkin’s lymphoma, ESRD, Osteoporosis, HTN |
| 16 | NRAS Q61K | 70 | M | C | Scalp/Trunk | Lung | 305 | 88 | 50 | Osteoporosis, BPH, Hypothyroidism, Hypercholesterolemia, arthritis, TIA, COPD, Cardiomyopathy, AF |
| 17 | BRAF V600E | 50 | M | C | Trunk | Brain, ABD (gastric), LNs (supraclavicular), SQ, IM | 210 | 17 | 5 | Anemia, hypothyroidism, HTN, HLP |
| 18 | BRAF V600E, NF1 splice site (2409+1G>A) | 42 | M | C | Lower Limb | N/A | 203 | 5 | 10 | N/A |
| 19 | QN | 36 | M | H | Lower Limb | LNs (pelvic, Left groin) | 213 | 1 | 0 | N/A |
| 20 | BRAF L485W | 65 | M | C | Lower Limb | Bone (popliteal), LNs (right leg) | 165 | 0 | 0 | Anxiety |
| 21 | BRAF V600K | 75 | M | C | Trunk | LNs (supraclavicular) | 209 | 20 | 5 | HTN, NMSK |
| 22 | BRAF V600K | 53 | M | C | Head | LNs (occipital, cervical) | 153 | 10 | N/A | Hypothyroidism, Hypopituitarism, Hypercholesterolemia, HTN, |
| 23 | BRAF V600E | 30 | F | C | Trunk | Brain, left scapular, adrenal, bone, LNs (mediastinal) | 138 | 1 | 0 | RA, DVT, TIDM |
| 24 | BRAF amplification | 64 | F | C | Head | N/A | 305 | 9 | N/A | Chronic lymphoid leukemia, IBS, arthritis, autoimmune hepatitis, hypothyroidism |
| 25 | BRAF V600E | 47 | M | H | Lower Limb | LNs (right leg) | 184 | 9 | N/A | Adrenal insufficiency, HTN, hypopituitarism, hypophysitis |
| 26 | BRAF D594N, NF1 E1206* | 47 | M | C | Face | Lung | 151 | 139 | N/A | HTN, hypercholesterolemia |
| 27 | BRAF G496R, NRAS Q61R | 52 | M | C | Trunk | LNs (right flank) | 105 | 8 | N/A | Ulcerative colitis, arthritis |
| 28 | QN | 76 | F | C | Trunk | - | 170* | 5 | N/A | Arthritis, hypothyroidism |
| 29 | HRAS G13V GOF | 52 | M | C | Face | LNs (Cervical) | 196 | 5* | 30 | Hypopituitarism, hypothyroidism, hypercholesterolemia, CAD, CABG, HTN, AF |
| 30 | BRAF V600E | 63 | F | C | Trunk | GI (gastric, colon), lung | 172 | 11 | 1 | Hypopituitarism, Hypothyroidism |
| 31 | BRAF V600E | 87 | M | C | Scalp | Liver, bone, bilateral neck | 359 | 14 | 100 | Hypercholesterolemia, HTN, TIIDM, arthritis, NMSK, CAD |
| 32 | KIT D816V, N882Y NF1 R135W, L62*, NRAS T50I | 76 | M | C | Trunk | LNs (Cervical), liver, bone | 281 | 106 | 40 | CHF, CAD, GERD, HTN, Hepatitis B, anemia, thrombocytopenia, pancytopenia |
| 33 | NRAS Q61K | 48 | M | C | Face | SQ (left leg) | 191 | 57 | 1 | BPH, CAD, HTN, Enterocolitis |
| 34 | NRAS Q61R, BRAF splice site (c.1400C>T), S467L | 20 | F | C | Face | Femoral, neck, chest | 193 | N/A | 0 | Lumbar radiculopathy |
| 35 | BRAF V600E | 48 | M | C | Trunk | Lung, subpectoral, LN (axilla), SQ | 139 | 13 | 1 | Panhypopituitarism |
| 36 | c-KIT V560E | 72 | F | C | Lower Limb | SQ (vulva), bone (spine), lung | 199 | 4 | 0 | Neutropenia, DVT, Leukopenia, monoclonal gammopathy, AF, HTN, PE, arthritis, HLP, asthma, GERD, Sjogren’s syndrome |
| 37 | NRAS Q61R mutation | 65 | F | C | Upper Limb | Lung, SQ (right arm) | 168 | 24 | 35 | Ulcerative colitis, DVT, Hypothyroidism, HTN |
| 38 | HRAS G13V | 75 | F | A | Upper Limb | LNs (axilla) | 181 | 1 | 1 | RA, Anemia |
| 39 | NRAS Q61K | 53 | M | C | Lower Limb | Brain, bone, liver, LNs (retroperitoneal), spleen, GI | 591 | 5* | 30 | DVT, HLP |
| 40 | BRAF V600E | 45 | M | C | Trunk | SQ, bone, lung, LNs (left axilla) | 176 | N/A | 51 | Adrenal insufficiency, arthritis, hypopituitarism, ulcerative colitis, arthritis, HTN, HLP |
| 41 | BRAF V600K | 51 | F | C | Trunk | LNs (left axilla) | 109 | 78 | 90 | Celiac disease, NMSK |
| 42 | KRAS G12V | 58 | M | H | Lower Limb | Liver, bone | 136 | 2 | N/A | Anemia, HTN, TIIDM |
| 43 | QN | 62 | M | C | Trunk | N/A | 160 | 4 | 0 | Anxiety, AA |
| 44 | NF1 loss exons 9-58 | 46 | F | PI | Head | Lung, scalp (occipital), LNs (cervical) | 138 | 4 | 0 | N/A |
| 45 | BRAF V600E | 25 | M | C | Trunk | LNs (right axilla), right adrenal gland, bone | 161 | 5 | N/A | Ulcerative colitis |
| 46 | KRAS K117N, BRAF N581H | 92 | M | C | Trunk | Bone | 280 | 26 | 0 | Hashimoto’s thyroiditis, ulcerative colitis, TIIDM, AF, CHF |
| 47 | RAF1-MAD1L fusion | 43 | M | C | Head | LNs (cervical), liver, bone | 240 | 19 | 10 | Arthritis |
| 48 | BRAF V600E | 68 | F | C | Lower Limb | SQ (leg), LNs (inguinal, iliac), bone | 159 | 10 | 1 | Hypothyroidism, HTN, bronchitis, |
| 49 | NRAS Q61H amplification | 47 | M | C | Trunk | Brain, LNs (left axilla, hilum) | 144 | 21 | 0 | DVT, ulcerative colitis, RA, panhypopituitarism, HTN |
| 50 | NRAS Q61K | 59 | F | C | Lower Limb | Bone, brain, lung, LNs (retroperitoneal) | 551 | 2 | 5 | Anxiety, interstitial pneumonitis |
| 51 | BRAF V600E | 65 | F | C | Lower Limb | SQ (leg) | 196 | 8 | 10 | Neuropathy, hypothyroidism, TIIDM, immune hepatitis |
| 52 | NRAS Q61R | 48 | M | C | Trunk | LNs (right axilla, mediastinal, and bilateral hilar) | 184 | 15 | N/A | Ulcerative colitis, HTN, RA, AA, MDD, psoriasis, HLP |
| 53 | QN | 36 | M | C | Head | SQ, LN (cervical) | 171 | 1 | 10 | Panhypopituitarism, arthritis |
| 54 | BRAF V600K | 71 | F | C | Trunk | LN (axillary), pituitary | 169 | 11 | 60 | Ulcerative colitis, HTN, MDD, arthritis, hypopituitarism |

UPN, unique patient number; QN, quadruple negative (no BRAF, NRAS, NF1, or KIT mutations identified); F, female; M, male; C, Caucasian; H, Hispanic; N/A, results not available; Y, yes; N, no; primary site; SQ, subcutaneous; LN, lymph node; ABD, abdomen; IM, intramuscular; unk, unknown; Ind, indeterminate; HCL, hypercholesterolemia; HTN, hypertension; TIIDM, Type 2 diabetes; CAD, coronary artery disease; AF, atrial fibrillation; BPH, benign prostatic hyperplasia; GERD, gastroesophageal reflux disease; COPD, chronic obstructive pulmonary disease; DVT, deep vein thrombosis; RA, rheumatoid arthritis; MDD, major depressive disorder; TIA, transient ischemic attack; AA, aortic aneurysm; NMSK, non-melanoma skin cancer; CHF, congestive heart failure; CABG, coronary artery bypass graft; N/A, none.
