# Supplementary material for: A combination of low TMB and PD-L1 expression predict poor progression-free survival of metastatic melanoma patients treated with first-line ipilimumab plus nivolumab
Source: Front Immunol. 2026 Jan 29;17:1729883. doi: 10.3389/fimmu.2026.1729883 (PMC12894002; doi:10.3389/fimmu.2026.1729883)
Supplement: Supplementary file 6 [file Table2.docx]

**Table 2 Treatment characteristics and patient outcomes**

| **UPN** | **Mutation** | **CKI Regimen** | **Initial CKI doses** | **ORR** | **PFS**  **(mo)** | **OS**  **(mo)** | **CKI toxicity** | **Current status** |
| --- | --- | --- | --- | --- | --- | --- | --- | --- |
| 1 | QN | ALT | 4 | CR | 11.4 | 11.4 | Rash | AWD |
| 2 | NRAS Q61K | ALT | 3 | CR | 2.3 | 17.6 | Rash | DOD |
| 3 | NRAS Q61L | STD | 4 | CR | 71.8 | 71.8 | Colitis, hyperthyroidism | NED |
| 4 | QN | ALT | 4 | PR | 16.7 | 19.6 | None | DOD |
| 5 | KRAS G12D, NF1 R440*, NF1 splice site (5609+1G>A) | STD | 2 | CR | 5.3 | 5.3 | Nausea, vomiting, diarrhea, colitis, fevers, chills | NED |
| 6 | BRAF V600E | ALT | 4 | CR | 3.3 | 19.0 | Diarrhea, pruritic | NED |
| 7 | BRAF V600E | ALT | 4 | CR | 29.1 | 41.4 | None | DOD (non-melanoma*) |
| 8 | BRAF V600E | ALT | 4 | PD | 0.7 | 8.9 | Arthritis | DOD |
| 9 | BRAF V600K, NRAS G60R | STD | 4 | CR | 70.9 | 70.9 | Pruritic, rash | NED |
| 10 | NRAS Q61L | ALT | 4 | SD | 5.5 | 40.0 | Diarrhea, colitis, GI hemorrhage | NED |
| 11 | HRAS G13R | ALT | 4 | CR | 23.6 | 24.0 | Pruritic, rash, arthritis | DOD (non-melanoma*) |
| 12 | BRAF V600E | ALT | 4 | SD | 16.2 | 16.9 | Neuropathy, xerostomia | NED |
| 13 | NF1 R440*, NF1 Q1174* | ALT | 3 | CR | 43.6 | 43.6 | Pruritic, rash | NED |
| 14 | QN | ALT | 3 | SD | 3.4 | 3.5 | None | AWD |
| 15 | Nonfunctional BRAF, NF1 splice site (c.2990+2T>A), R1276* | - | 4 | PD | 1.6 | 10.6 | Pruritic, rash | DOD (Unknown Cause) |
| 16 | NRAS Q61K | ALT | 2 | CR | 47.7 | 47.7 | Pruritic, rash | NED |
| 17 | BRAF V600E | ALT | 4 | CR | 26.6 | 26.6 | None | NED |
| 18 | BRAF V600E, NF1 splice site (2409+1G>A) | ALT | 4 | CR | 18.5 | 18.5 | None | NED |
| 19 | QN | ALT | 4 | PD | 3.0 | 23.0 | None | DOD (Unknown Cause) |
| 20 | BRAF L485W | STD | 4 | PD | 3.9 | 12.4 | None | DOD (Unknown Cause) |
| 21 | BRAF V600K | ALT | 3 | PR | 1.7 | 4.4 | None | DOD (Unknown Cause) |
| 22 | BRAF V600K | ALT | 4 | CR | 34.7 | 34.7 | Hypopituitarism, fatigue, headaches, constipation | NED |
| 23 | BRAF V600E | STD | 5 | PD | 1.2 | 25.6 | TIDM, arthralgia, rash | AWD |
| 24 | BRAF amplification | STD | 2 | CR | 14.7 | 14.7 | Arthralgia, rash, hypothyroid | NED |
| 25 | BRAF V600E | STD | 4 | CR | 29.4 | 29.4 | Fatigue, presyncope, anorexia, nausea, uveitis, hypopituitarism | NED |
| 26 | BRAF D594N, NF1 E1206* | STD | 4 | CR | 68.5 | 68.5 | None | NED |
| 27 | BRAF G496R, NRAS Q61R | ALT | 4 | PR | 6.5 | 12.2 | Colitis, diarrhea | DOD |
| 28 | QN | STD | 4 | CR | 19.7 | 19.7 | Hyperthyroidism | Unk |
| 29 | HRAS G13V GOF | ALT | 4 | PR | 0.7 | 17.2 | Diarrhea, fever, fatigue | AWD |
| 30 | BRAF V600E | ALT | 4 | CR | 46.6 | 46.6 | Pruritic, rash, headaches, fatigue, hypopituitarism | NED |
| 31 | BRAF V600E | ALT | 3 | PD | 2.2 | 2.3 | Arthralgia, fatigue | DOD (Unknown Cause) |
| 32 | KIT D816V, N882Y NF1 R135W, L62*, NRAS T50I | ALT | 4 | CR | 48.4 | 48.4 | Pruritic, rash | NED |
| 33 | NRAS Q61K | ALT | 4 | CR | 19.6 | 58.4 | Diarrhea | NED |
| 34 | NRAS Q61R, BRAF splice site (c.1400C>T), S467L | STD | 4 | SD | 6.4 | 6.4 | Anorexia, mucositis | NED |
| 35 | BRAF V600E | ALT | 3 | CR | 9.5 | 45.1 | Hypophysitis, hypopituitarism | AWD |
| 36 | c-KIT V560E | ALT | 4 | PR | 6.6 | 16.5 | None | DOD (non-melanoma*) |
| 37 | NRAS Q61R mutation | ALT | 4 | CR | 17.0 | 17.0 | Diarrhea, pruritic | NED |
| 38 | HRAS G13V | ALT | 4 | PR | 4.2 | 7.3 | Pruritic, rash | Unk |
| 39 | NRAS Q61K | ALT | 3 | PD | 1.3 | 2.1 | None | DOD (Unknown Cause) |
| 40 | BRAF V600E | STD | 4 | CR | 101.6 | 101.6 | Diarrhea, pancreatitis, panhypopituitarism, ulcerative colitis | NED |
| 41 | BRAF V600K | ALT | 4 | CR | 36.8 | 36.8 | Cramps, nausea, vomiting | NED |
| 42 | QN | ALT | 4 | PR | 3.2 | 13.8 | None | DOD (Unknown Cause) |
| 43 | BRAF V600E | ALT | 4 | PD | 3.0 | 40.8 | None | NED |
| 44 | BRAF V600E | STD | 4 | PD | 2.3 | 8.2 | Diarrhea, rash | DOD (Unknown Cause) |
| 45 | KRAS K117N, BRAF N581H | STD | 4 | CR | 5.7 | 71.4 | Chills, fever, diarrhea | NED |
| 46 | RAF1-MAD1L fusion | STD | 3 | PR | 4.8 | 4.8 | Diarrhea, rash | AWD |
| 47 | BRAF V600E | ALT | 4 | CR | 36.8 | 36.8 | None | NED |
| 48 | NRAS Q61H amplification | ALT | 2 | PD | 0.9 | 1.6 | None | DOD (Unknown Cause) |
| 49 | NRAS Q61K | STD | 4 | CR | 20.6 | 20.6 | Arthritis, diarrhea, pruritic, rash | NED |
| 50 | BRAF V600E | STD | 4 | PD | 5.0 | 6.6 | Nausea, vomiting | Unk |
| 51 | NRAS Q61R | STD | 3 | CR | 25.0 | 25.0 | Hypothyroidism, pruritic, rash, TIDM | NED |
| 52 | QN | ALT | 2 | CR | 41.8 | 41.8 | Diarrhea, fatigue, GI hemorrhage, headaches, pancolitis, presyncope, rash | NED |
| 53 | BRAF V600K | STD | 2 | PD | 10.6 | 27.2 | Arthralgia | NED |
| 54 | QN | ALT | 4 | CR | 38.4 | 38.4 | Colitis, diarrhea, hematochezia, hypopituitarism, fatigue, rash | NED |

UPN, unique patient number; BRAF F/R, BRAF gene fusion or rearrangement; QN, triple negative (no BRAF, NRAS, NF1, or KIT mutations detected); CKI, checkpoint inhibitor; I, ipilimumab; N, nivolumab; SND, standard treatment (3 mg/kg I, 1 mg/kg N); ALT, alternate treatment (1 mg/kg I, 1 mg/kg N); ORR, objective response rate; PFS, progression-free survival; OS, overall survival; OR, overall response; PD, progressive disease; CR, complete response; CR (TT), complete response due to addition of targeted therapy; SD, stable disease; PR, partial response; NED, no evidence of disease; DOD, died of disease; COD, cause of death; AWD, alive with disease
